# Telenurses’ work environment - Relationships between working conditions, remote work from home or not and the outcomes job satisfaction, burnout and thriving

**DOI:** 10.1177/20552076261450322

**Published:** 2026-05-27

**Authors:** Annica Björkman, Karin Myrberg, Anna Carin Wahlberg, Maria Engström

**Affiliations:** 1 Department of Health and Caring Sciences, 3485 University of Gävle, Gävle, Sweden; 2 Department of Neurobiology, Caring Sciences and Society, 27106 Karolinska Institute, Stockholm, Sweden

**Keywords:** remote work, telenursing, teleworking, work environment, job satisfaction

## Abstract

**Objective:**

To examine relationships between working conditions, remote work and the outcomes job satisfaction, thriving and burnout among nurses engaged in telenursing.

**Methods:**

The study had a cross-sectional and correlational design. Questionnaire data from 182 telenurses were collected during 2023 using a web-based survey and analysed using multiple linear regression models.

**Results:**

For job satisfaction and thriving, the regression models revealed that nurses who rated better working conditions and worked remotely (fully or partially) scored statistically significantly higher in job satisfaction and thriving. In addition, older age was statistically significantly associated with better job satisfaction in the model. For burnout, remote work was non-significant; however, better working conditions and older age were statistically significantly associated with lower burnout scores. The three models’ explanatory power (R^2^ Adjusted) for each outcome were 39% of the variance in job satisfaction, 38% in thriving and 47% in burnout.

**Conclusion:**

Remote work among telenurses is related to higher job satisfaction and thriving compared to solely office-based work. Given the cross-sectional design, causal interpretations cannot be made.

## Introduction

Remote work, also defined as telework is not a new concept, however it garnered substantial attention during the COVID-19 pandemic. Although the term may vary and definitions can differ slightly, remote work is generally understood as a work arrangement in which individuals perform their job tasks outside the conventional office setting, utilizing digital technologies as necessary to facilitate their work.^1,2^ Following the COVID-19 pandemic, remote work has expanded across multiple sectors including the nursing profession. Partly because of the COVID-19 pandemic, registered nurses (RN´s) employed within the Swedish national healthcare service 1177 were broadly offered the opportunity to work from home. The rationale behind this new mode of working was twofold: to reduce the risk of viral transmission in the workplace and to ensure the availability of sufficient staffing resources. During this time period it was common for personnel to be placed in quarantine after contact with individuals suspected of being infected with COVID-19.^3^ Despite the lifting of restrictions following the decline of the COVID-19 pandemic, remote work has remained an alternative work arrangement for some nurses within the Swedish Healthcare Direct (SHD) the national telenursing service in Sweden.

Many countries have introduced national telenursing services as a first portal to healthcare to optimize the use of health-care resources. Different approaches are used in Europe e.g., telephone triage and several other initiatives focusing on self-advice tools, triage preparation, and chatbots.^4^ Telenursing is the provision of nursing care via telephone, video, and/or chat, involving triage, advice, and nursing interventions provided by a registered nurse (RN), hereafter defined as telenurses.^5^ The contact with the telenursing service usually results in exchange of health information about bodily functions, symptoms, risks and/or medications ^5,6^ as well as advice to visit an emergency department, to see a general practitioner and other healthcare providers, or to perform self-care.^7^

Evidence on the cost-effectiveness of telenursing indicates that it is comparable to traditional care,^8^ has the potential to reduce the number of immediate physician visits, and does not lead to an increase in emergency department visits.^9^ Data from Sweden suggest that telenursing contributes to a shift in healthcare visits from secondary to primary care, indicating that telenursing increases system efficiency.^10^ The service has patient satisfaction levels comparable to or higher than traditional care.^11^ SHD is available around the clock, is free of charge and receives approximately 11,000 calls per day. Telenurses working at SHD are typically experienced specialists trained in the Dialogue process—a structured communication framework applied during calls and chats to systematically assess the situation and collect sufficient information for safe clinical decision-making.^5^

Telenurses work environment have been described as demanding work resulting in cognitive fatigue but also being able to be an expert in nursing care.^12,13^ In this study, the term *work environment* is used as an overarching concept encompassing the nature of work, its content, and the context in which it is performed. Theoretically it comprises three components: physical, psychological, and social work environments. The latter two are often combined under the concept of the psychosocial work environment.^14,15^ The physical work environment includes factors such as lighting, noise, air quality, and ergonomic aspects like desks and chairs.^15^ The psychosocial work environment is more complex, involving elements such as job control, demands, leadership, opportunities for influence, managerial and collegial support, and work–life.^14,16^

Nurses work environment has been found related to the outcome of care, patient safety,^17,18^ nurse job satisfaction and negative aspects such as stress symptoms and burnout^19,20^ within other fields of nursing. Job satisfaction measures workers’ contentment with their job, whether they like the job or individual aspects or facets of jobs, such as nature of work or supervision. Job satisfaction can be measured in cognitive (evaluative), affective (or emotional), and behavioral components.^21^ Burnout has been associated with absenteeism, poor staff retention, low staff morale, poor performance, disturbed sleep, poor health outcomes and all-cause mortality for the respondent, but also the clinical outcomes of their patients.^22^ Burnout is a central outcome within the Job Demands–Resources (JD-R) theory and has been linked to absenetism, difficulties in staff retention, impaired performance, sleep disturbance and adverse health outcomes.^23^ Hence the JD-R theory^24,25^ might provides a useful framework for understanding how work-related demands and available resources shape employee well-being, performance, and the development of job strain.

Thriving has been found related to outcomes such as employee health, wellbeing, job performance, attitudes, and development,^26^ and is described as both a sense of vitality and a sense of learning for the employee at work.^27^ In a previous study, we found that good access to structural empowerment increased telenurses’ thriving, which in turn improved work-personal life benefits, job performance, and decreased stress symptoms and turnover intentions^28^

Previous studies conducted in contexts other than nursing care, focusing on remote work and employee well-being, generally indicate that remote work has been foun related to beneficial for employees. Benefits include greater autonomy that may increase job satisfaction and decrease emotional exhaustion. However, remote work may also relate to negative effects, such as isolation that can decrease job satisfaction and performance.^29,30^ Australia’s challenging geographical conditions have positioned it as an early adopter of various telehealth and tele care delivery models. Research suggests that nurses during remote work exhibit higher productivity, take fewer sick leave days, and experience lower attrition rates. Reported advantages include flexible working hours and reduced commuting, while no disadvantages were identified.^31^

Although remote work has been widely examined in non-clinical sectors often highlighting benefits such as increased autonomy, flexibility, and reduced commuting^32,33^ these findings may not directly translate to clinical contexts. Telenursing involves relational, emotionally demanding,^12,34^ and high-stakes decision-making, where nurses assess patients’ needs, provide guidance, and make triage decisions without physical examination. These conditions differ substantially from knowledge-based office work typically represented in the remote-work literature. Therefore, it remains unclear whether the established patterns regarding remote work also apply to telenurses, whose work is simultaneously cognitively complex, time-pressured, and safety-critical. This contrast underscores the importance of investigating remote work specifically within telenursing.

The JD-R theory^24,25^ posits that employee well-being is shaped by the balance between job demands and job resources. Within the framework of nursing, remote work may introduce specific demands—such as professional isolation or blurred boundaries—and resources, including increased autonomy, flexibility, and reduced interruptions. Applying the JD-R theory provides a theoretical rationale for examining how remote versus office-based work conditions relate to telenurses’ job satisfaction, thriving and burnout.

To date, little is known about how nurses with the option to work remotely from home perceive their work environment. While remote work in other sectors has been linked to benefits such as increased flexibility and productivity, nursing differs substantially due to its relational nature, high cognitive demands, and reliance on structured communication processes. This raises important questions about whether remote work is related to critical outcomes such as job satisfaction, thriving, and burnout. Understanding these relationships is essential, as they might have implications not only for employee well-being and retention but also for the quality and safety of patient care as these outcomes, in turn, have been related to patient outcomes.^17,35,36^ Addressing this gap, the present study aims to examine relationships between work environment conditions, remote work, and key occupational outcomes among nurses engaged in telenursing.

## Methods

### Study design

The study had a cross-sectional and correlational design.

### Study setting and sample

All regions in Sweden connected to the national network 1177 (n=21) were invited to participate in the study, 14 out of 21 agreed to participate following internal administrative decisions within their respective healthcare organizations. Differences between participating and non-participating regions are not systematically documented; however, administrative priorities and resource availability at the time of recruitment may have influenced participation. All telenurses working within these 14 regions (n=465) were invited to participate in a survey about their working conditions (more specific aspects related to telenursing and more general aspects such as working remotely yes or not at all i.e. working slolely at the office) and wellbeing. In total, 465 telenurses were invited, and the response rate was 39.1% (n=182). Most participants were female (93.4%) and 45% had permission from their responsible manager to work remotely and also worked fully or partially remotely. The mean age was 53 years and the mean working time within telenursing were 7.5 years. See Table 1 for participant characteristics.

**Table 1.** Participant characteristics.

Age, year, mean (SD)	53.1 (11.1)
Working time telenurse, year, mean (SD)	7.5 (8.4)
Working time as a registered nurse in total, year, mean (SD)	24.8 (12.2)
Sex, n (%)	
male	10 (5.5)
female	170 (93.4)
Remote work, n (%)	
yes (fully or partially)	82 (45.1)
no	86 (47.3)

Standard deviation (SD). When sum does not add to 182 there are internal missing data.

### Data collection

Data were collected using a structured web-based survey during sent out during the period November 2023 until Januari 2024 and two reminders were sent. The questionarire compromised the following sections, used in the present study.

• *Socio-Demographic Information*; background variables e.g. age, gender, workingtime as telenurse, working time as registred nurse in total and opportunity to work remotely.
• Job satisfaction was measured with the *Brief Index of Affective Job Satisfaction.*^37^ The instrument consists of four items and the response alternatives range from 1) Strongly disagree to 4) Strongly agree. The scale mean is calculated, and higher scores represent higher job satisfaction. Validity^37^ and reliability, measured as internal consistency, have been reported to be good.^20^ 84 in the present study.
• Thriving was measured with the 10-item *Thriving scale*^27,38^ with response alternatives ranging from 1) Disagree strongly to 7) Agree strongly. The scale mean is calculated and higher scores represent higher thriving. The instrument consists of two factors, vitality 5 items and learning 5 items. Construct validity and internal consistency have been found good^38,39^ Cronbach’s Alpha in the present study 0.91.
• Burnout was measured with the factor Personal Burnout from the Safety, Communication, *Operational Reliability and Engagement (SCORE) questionnaire*^23^ with response alternative from 1) Disagree strongly to 7) Agree strongly. The mean of the raw scores for the included items is calculated and then subtracted by 1 and multiplied by 25. Higher scores represent higher burnout. Validity and reliability of the SCORE questionnaire have been found good.^23^ Cronbach’s Alpha in the present study 0.91.
• Working conditions were measured with the *Telenursing Working Condition (TWC)* scale.^13^ The scale consists of 14 items and 3 factors (Facilitating conditions, Management and colleagues and Barriers), response alternatives ranging from 1) Not at all to 5) To a very high extent. The scale and factors’ mean scores are calculated, and higher scores represent better conditions. Example of facilitating conditions items; *“focus on one caller at a time”, “access to structure and support through the decision-support system” and “work in a calm and pleasant environment”.* Example of Mangement and colleagues items; “*support from management”* and *“opportunity to discuss and reflect with colleagues”.* Example of Barriers items; *“lack of opportunity to influence my work” and “periodically monotonous work”.* In the present study total scale was used. The Telenurse Working Condition Scale^13^ was developed in 2016 in collaboration with telenurses in Sweden to provide a domain-specific measure of working conditions within telenursing. The study was based on a modified Delphi approach, incorporating qualitative and quantitative data sequentially across three phases. In Phase I, data were generated through semi-structured interviews. In Phases II and III, data were subsequently collected using a web-based survey. Although the original validation data is unpublished, the scale has demonstrated acceptable psychometric properties, including construct validity and internal consistency. In the present study, the scale showed satisfactory reliability, with a Cronbach’s alpha of 0.82. The development of the scale in Sweden supports its relevance and applicability in the Swedish telenursing context. See Supplementary file 2 for complete questionnaire.
• To dichotomize the nurses into remote work yes or no we used two questions from the survey. Remote work “yes” could then be both fully or partially remotely. The questions we used were “Do you have the option of remote work?” Reponses were Yes or No. If a Yes here we checked the question “How do you usually divide your working hours between the office and remote work?”. Responses were free text/number in relation to “Number of days in the office/week” …. and “ Number of days remotely/week” …. A combination of these two were used to get the dichotomized variable remote work yes or no. In the Supplementary file 1 there is a table describing how many days the nurses reported that they usually worked remotely/week. Most nuses reported that they usually worked two days remotely (n=18, 22 %) and three days remotely (n=17, 20.7 %).

### Data analysis

Data were analysed using IBM SPSS Statistics Version 30. To compare groups, Mann-Whitney U-test was used and for correlational analyses we used Spearman’s rho and Multiple Linear Regression Models. No correction for multiple comparisons was used. In the Multiple Linear Regression Models the outcomes were Job satisfaction, Thriving and Burnout. Predictors were remote work yes (fully or partially) or no not at all, and telenursing working conditions. In all models we controlled for age. Sex was not controlled for as there were only 10 males. Listwise deletion of cases was used when there were missing data for any of the included variables, meaning that the analyses were based on n 162 for job satisfaction, 163 for thriving, and 163 for burnout. For variables in all models, variance inflation factor (VIF) values were less than 1.1 indicating no problem with multicollinearity. Visual inspection of residuals, such as histograms, and Normal Q-Q Plots indicated no problems with the normality of data. For regression analyses, N ≥50+8m participants are needed, where m is the number of predictors in the regression. In our study, we had 182 telenurses and we had four predictors in the regression, i.e., a need for 82 participants for the statistical analysis.

### Research ethics

All employees working within the 14 participating regions of the national 1177 service were invited to participate in the study via an email distributed by the reseachers. The email contained written information about the purpose and procedures of the study. Individuals who wished to participate were instructed to click on a link to enter the web-based questionnaire provided at the end of the email. The questionnaire started with the same written study information, along with a statement clarifying that participation was voluntary. The information in the questionarie stated that by completing and submitting the questionnaire, participants provided their written informed consent to take part in the study. This approach was approved by the Swedish Ethical Review Authority, approval number Dnr 2023-01378-02.

## Results

### Bivariate correlations between study variables

The results revealed statistically significanbetween ‘Telenursing working conditions’ and all outcome variables, Spearman’s rho ranging from .569 to -.695, and between age and Job satisfaction and Burnout, see Table 2.

**Table 2.** Relationships between study variables, Spearman’s rho.

​	Mean (SD)	TWC	JS	Thriving	Burnout
Telenursing working conditions	5.2 (.8)	​	​	​	​
Job satisfaction (JS)	3.5 (.8)	.569***	​	​	​
Thriving	5.4 (1.0)	.574***	.662***	​	​
Burnout	22.7 (23.8)	-.695***	-.498***	-.437***	​
Age	53.1 (11.0)	.191*	.185*	.024	-.335***

Abbrevations: SD Standard deviation, JS Job satisfaction *p<0.05, **p<0.01, ***p<0.001.

### Comparing the groups remote work or not

Among nurses dichotomized to remote work they could work fully or partly while nurses in the no-group worked solely in the office. The results revealed statistically significantly higher scores in Job satisfaction for the group remote work (fully or partially) compared to those working only at the office. For Thriving, Burnout and ‘Telenursing working conditions’ the results were non-significant, see Table 3.

**Table 3.** Job satisfaction, Thriving, Burnout and ‘Telenursing working conditions’ compared between the groups Remote work (fully or partially) and Not remote work, Mann-Whitney U-test and effect size (*r*).

​	Remote work	Not remote work	​	Effect size *r*^*1*^
Variables	Mean (SD)	Mean (SD)	p-value	​
Telenurse Working Condition, n=82 remote work/84 no remote work	5.1 (0.8)	5.2 (0.7)	.781	-0.02
Job satisfaction, n=82/83	3.7 (0.9)	3.4 (0.8)	.026	0.17
Thriving, n=81/85	5.5 (1.0)	5.3 (0.9)	.061	0.14
Burnout, n=81/85	23.3 (24.0)	23.4 (23.5)	.720	-0.03

Abbrevations; SD standard deviation. ^1^ Effect size was computed for Mann-Whitney U Test using z test statistic.^40^ According to Funder and Ozer (2019), an r of .10 indicates a small effect and .20 a medium effect.^41^

### Multiple linear regression models - Outcomes job satisfaction, thriving and burnout

For Job satisfaction, the results from the multiple linear regression model revealed that nurses who worked remotely, and rated better ‘Telenursing working conditions’, and were older scored statistically significantly higher on Job satisfaction. The model’s explanatory power (R^2^ adjusted) was 39%. For Thriving, the statistically significant variables in the model were also remote work and ‘Telenursing working conditions’ and the model’s explanatory power was 38%. For Burnout, remote work was non-significant in the model; however, higher scores on ‘Telenursing working conditions’ and older age were statistically significantly associated with lower scores on burnout and the model’s explanatory power was 47% (Table 4). For all three models the predictor ‘Telenursing working conditions’ had the highest standardized beta coefficients (.548 Job satisfaction, .618 Thriving and -.623 Burnout). Remote work when statistically significant in the models of Job satisfaction and Thriving had standardized beta coefficient .168 and .131 respectively (Table 4).

**Table 4.** Multiple linear regression models of Job satisfaction, Thriving and Burnout.

Predictors	Job satisfaction (n=162)		Thriving (n=163)		Burnout (n=163)	
	R^2^ .40/Adj R^2^ .39		R^2^ .39/Adj R^2^ .38		R^2^ .48/Adj R^2^ .47	
​	β	p-value	β	p-value	β	p-value
Remote work: no=0/yes=1	.168	**.007**	.129	**.039**	-.019	.747
Telenursing working conditions	.547	**<.001**	.615	**<.001**	-.623	**<.001**
Age	.189	**.003**	-.032	.608	-.205	**<.001**

Abbrevations: β Standardized beta coefficient, bold values indicate statistically significant results. Listwise deletion of cases was used for missing data for any of the included variables, meaning that the analyses were based on n 162 for job satisfaction, 163 for thriving, and 163 for burnout.

## Discussion

This study is, to our knowledge, the first to examine relationships between telenurses’ working conditions, whether they work remotely from home (fully or partially) or not, and the outcomes of job satisfaction, Thriving, and Burnout. In summary, the study found that telenurses with opportunity to remote work reported significantly higher job satisfaction and thriving when other variables were hold constant (working condition and age). However, for burnout remote work or not were non-significant in the model. For all outcomes, telenursing working condition was statistically significant in the models and had the highest standardized beta coefficient. The models explained 38%, 39% and 47% of the variance in thriving, job satisfaction and burnout respectively.

There is research from other professional fields of relationships between remote work, working conditions and wellbeing that confirms our results.^42,43^ For working condition several studies have found positive relationship with staff wellbeing.^20,39^ However, for remote work this study adds to earlier research with adding the nursing context. Our results, emphasizes the importance of considering both the working conditions and if the nurses worked remotely or not in understanding work wellbeing.

In addition to the main findings of remote work, the study reveals that favourable work conditions among telenurses are strongly related with higher job satisfaction, a sense of thriving, and lower burnout. Relationships between Job satisfaction and Thriving are well-documented in existing literature,^44,45^ although more sparsely among telenurses. The strong association between working conditions and well-being observed in the present study is consistent with evidence from other high-stakes nursing contexts. Recent research among nurses in neonatal care units demonstrates that favourable perceptions of the work environment are closely linked to key components of professional quality of life, including lower burnout levels.^46^ Similarly, Ayed et al.^47^ reported significant negative correlations between core aspects of the practice environment—such as participation in organizational affairs and strong foundations for high-quality care—and burnout among intensive care nurses. Together, these findings reinforce the interpretation that it is the quality of the work environment, rather than the physical work location, that plays the decisive role in mitigating burnout risk. This cross-contextual evidence supports the relevance of the JD-R theory for understanding the telenursing context and underscores the importance of organizational support structures in safeguarding nurses’ well-being across different clinical settings. Understanding these relationships can also inform strategies to enhance the work environment for telenurses, potentially leading to interventions aimed at increasing job satisfaction and reducing burnout such as strengthening opportunities and time for peer support, discussions and reflection. This could be scheduled as there is limited time today when everyone is busy with tele encounters.

Age has weak positive relationships with work conditions and job satisfaction, and a moderate negative with burnout. Older telenurses tend to perceive their work environment more positively and report higher job satisfaction. Furthermore, there is a moderate negative relationship between age and burnout, suggesting that older telenurses tend to experience lower levels of burnout. This finding is consistent with previous observations in large populations of nurses.^48^ Potential reasons for these relationships might include greater experience and more well-developed coping mechanisms among older telenurses. These relationships highlight the importance of considering age and its relationships with workplace perceptions and well-being.^49^ It has been previously described that nurses, particularly younger women, who had the opportunity to work remotely during the pandemic experienced less stress, less burnout, and more satisfaction while maintaining work efficiency.^50^ This apparent discrepancy between earlier findings and our results likely reflects key contextual differences. Prior studies reporting reduced burnout among nurses working remotely during the COVID-19 pandemic^50^ were conducted in acute-care settings where remote work replaced highly demanding in-person clinical duties during a crisis. In contrast, telenurses perform similar cognitive and communicative tasks regardless of work location, meaning that remote work does not fundamentally reduce core job demands. The present study was conducted in a post-pandemic context characterized by greater operational stability, which may further explain why remote work did not translate into lower burnout in this cohort. Although remote work was not significant in the burnout regression model, this divergence from the findings for job satisfaction and thriving is theoretically understandable. Job satisfaction and thriving reflect positive, motivational aspects of well being that respond readily to increases in job resources such as autonomy or flexibility associated with remote work. In contrast, burnout is more strongly shaped by persistent job demands and strain related processes, as described in the JD-R theory.^24,25^ Existing research also shows that burnout is closely linked to high job demands, technology frustration, and insufficient structural or psychological support,^42,43^ factors that remote work alone may not sufficiently buffer. Thus, while remote work may enhance positive experiences, it may not substantially mitigate the broader demand related mechanisms that drive burnout among telenurses. Despite the non-significant relationship between the possibility of remote work and burnout in the present study, the overall results suggest that remote work may relate to other benefits that could reduce burnout. However, telenursing have been reported as emotionally demanding^12,31^ and high-stakes decision-making wich might explain the non-significant burn-out scores. Remote work does not change these core job demands, and may therefore have limited impact on reducing burnout.

Current evidence demonstrates that telenursing hold numerous benefits for care^5^ and for cost and efficiency optimization.^10,49^ Telenurses in primary care lead to reduced in-patient consultations and may contribute to easing workload burdens in other specialties, such as specialized care.^51^ However, this effect is of limited relevance for telenurses experiencing high workload, communicative demands, and feelings of being constantly monitored.^12,52^ Previously described benefits of telenurses include a sense of autonomy, which is generally linked with lower burnout,^53^ and a sense of being experts in nursing care.^12^ Additionally, support from colleagues has been mentioned as a significant benefit.^13^ In remote work you could assume that support from colleagues could be less common, however our results does not indicate this. Given the results from the present study, managers should strive to increase thriving while simultaneously encouraging collegial support, which might also be achieved at a distance.^42^ A deeper understanding of the relationships described in this study can guide healthcare organizations in tailoring work conditions for telenurses. However, to keep in mind is that remote work was related to Thriving when the other variables were hold constant (i.e. working conditions and age).

### Strengths and limitations

The major limitations of the study are the cross-sectional design, how remote work was measured and response rate. The cross-sectional design does not allow causality conclusions. For example, do satisfied nurses choose remote work, or does remote work cause satisfaction? Furthermore, the absence of data on the proportion of time that telenurses spent working remotely, no dose-response measure, limits the conclusions. Even though they responded with their usual/approximate number of days/week of remote work, we do not know how much they worked in total in telenursing for those who worked part time. It will be crucial for future studies to collect data on the proportion of time telenurses spend working remotely to explore whether there is an optimal level of remote work that maximizes job satisfaction and thriving. Another limitation is the response rate of 39%, which reduces generalizability. There might be potential non-response bias if those being more positive are the ones who have responded. As no information was available on non-respondents, potential non-response bias cannot be assessed. The sample characteristics are comparable to national data on telenurses^12,13^ which may mitigate concerns regarding representativeness. We used age as confounders, however, there might be other confounders that we did not measure. Still another limitation is that only ‘Personal burnout” from SCORE was used i.e., a single-factor burnout measure and thereby not measuring depersonalization/cynicism and professional efficacy. Strengths are the use of validated instruments with good internal consistency in the present study.

## Conclusions

Possibilities to remote work is a viable option to telenurses.

This study demonstrates that remote work among telenurses is associated with significantly higher levels of job satisfaction and thriving compared to solely office-based work when working conditions and age were hold constant, while no significant differences were observed for burnout. Telenursing working conditions are positively related to job satisfaction and thriving, and negatively to burnout.

## Implications and recommendations

The findings have several important implications. First, they highlight the critical role of working conditions in shaping positive occupational outcomes and suggest that remote work can be an effective strategy to enhance learning, vitality (thriving) and satisfaction. Second, the absence of differences in burnout challenges the assumption that remote work increases the risk of isolation or emotional strain, indicating that remote work can be implemented without compromising mental health. Future research should further explore which specific remote-work-related demands and resources are most salient for telenurses and how these factors interact over time in line with the JD-R theory.

Healthcare organizations should consider flexible work arrangements, including remote work, as part of their strategies to improve job well-being and retain staff. At the same time, it is essential to maintain strong support structures and access to necessary resources, to prevent potential social isolation. To operationalize strong support structures for remote telenurses, organizations may implement measures such as scheduled virtual peer-support meetings, structured online reflective practice sessions, remote-leadership training for managers, and clear protocols to prevent digital presenteeism (e.g., guidance on availability and breaks). These targeted practices can help sustain collegial connection, promote learning, and safeguard well-being when telenurses work remotely. Continuous monitoring of staff well-being through regular assessments of job satisfaction, thriving, and burnout is recommended to identify emerging challenges early. Finally, organizations should invest in reliable digital infrastructure and provide training to ensure that remote work functions smoothly and that technical barriers do not undermine its benefits.

## Supplemental material


Supplemental material - Telenurses’ work environment - Relationships between working conditions, remote work from home or not and the outcomes job satisfaction, burnout and thriving
Supplemental material for Telenurses’ work environment - Relationships between working conditions, remote work from home or not and the outcomes job satisfaction, burnout and thriving by Annica Björkman, Karin Myrberg, Anna Carin Wahlberg and Maria Engström in DIGITAL HEALTH.

Supplemental material - Telenurses’ work environment - Relationships between working conditions, remote work from home or not and the outcomes job satisfaction, burnout and thriving
Supplemental material for Telenurses’ work environment - Relationships between working conditions, remote work from home or not and the outcomes job satisfaction, burnout and thriving by Annica Björkman, Karin Myrberg, Anna Carin Wahlberg and Maria Engström in DIGITAL HEALTH.

## Data Availability

The data presented, aggregated data, during the current study are available from the corresponding author upon reasonable request. Signing a data sharing agreement will be necessary. Individual data are unavailable due to General Data Protection Regulations (GDPR) and in accordance with the ethics application.*
