# Supplementary material for: Telenurses’ work environment - Relationships between working conditions, remote work from home or not and the outcomes job satisfaction, burnout and thriving
Source: Digit Health. 2026 May 27;12:20552076261450322. doi: 10.1177/20552076261450322 (PMC13219931; doi:10.1177/20552076261450322)
Supplement: Supplemental material - Telenurses’ work environment - Relationships between working conditions, remote work from home or not and the outcomes job satisfaction, burnout and thriving [file sj-pdf-1-dhj-10.1177_20552076261450322.pdf]

Supplementary file 1

Table 1 Number of days working remotely/week among nurses reporting working remotely (n=82)

**Days working remotely/week**	**n**	**%**
1 day/week	13	15.9
2 days/week	18	22
3 days/week	17	20.7
4 days/week	13	15.9
5 days/week	16	19.5
6 days/week	0	
7 days/week	2	2.4
Missing *	3	3.7
Total	82	

*There were three missing, but these nurses had reported that they were 0 days at the office and thus dichotomized as remote work. Among those 86 nurses who responded solely office work 41 responded that they had the possibility to work remotely.

Table 2 Multiple linear regression models of Job satisfaction, Thriving and Burnout – Unstandardized beta coefficient and 95% Confidence Intervals (CIs) added compared to Table 4 in the manuscript

**Predictors**	**Job satisfaction (n=162)** R^2 .40/Adj R^2 .39			**Thriving (n=163)** R^2 .39/Adj R^2 .38			**Burnout (n=163)** R^2 .48/Adj R^2 .47		
	β	p-value	Unstandardized beta (95% CIs)	β	p-value	Unstandardized beta (95% CIs)	β	p-value	Unstandardized beta (95% CIs)
**Remote work:** no=0/ yes=1	.168	**.007**	.282 (.078;.486)	.129	**.039**	.256 (.013;.499)	-.019	.747	-.879 (-6.253;4.495)
**Telenursing working conditions**	.547	**<.001**	.579 (.448;.711)	.615	**<.001**	.765 (.610;.921)	-.623	**<.001**	-18.591 (-22.032;-15.151)
**Age**	.189	**.003**	.014 (.005;.024)	-.032	.608	-.003 (-.014;.008)	-.205	**<.001**	-.439 (-.685;-.193)

Abbreviations: β Standardized beta coefficient, TWC telenursing working conditions,

Bold values indicate statistically significant results. Listwise deletion of cases was used for missing data for any of the included variables, meaning that the analyses were based on n 162 for job satisfaction, 163 for thriving, and 163 for burnout.
