# Supplementary material for: Telenurses’ work environment - Relationships between working conditions, remote work from home or not and the outcomes job satisfaction, burnout and thriving
Source: Digit Health. 2026 May 27;12:20552076261450322. doi: 10.1177/20552076261450322 (PMC13219931; doi:10.1177/20552076261450322)
Supplement: Supplemental material - Telenurses’ work environment - Relationships between working conditions, remote work from home or not and the outcomes job satisfaction, burnout and thriving [file sj-pdf-2-dhj-10.1177_20552076261450322.pdf]

Telenursing Working Condition (TWC)

Working conditions were measured with the *Telenursing Working Condition (TWC)* scale (13). The scale consists of 14 items and 3 factors (Facilitating conditions, Management and colleagues and Barriers), response alternatives ranging from 1 to 5).

	Not at all	Almost not at all	To a small extent	To some extent	To a fairly large extent	To a large extent	To a very large extent
1. I can focus on one caller at a time							
2. I work in a calm and pleasant environment							
3. I can adjust my workstation							
4. I have a stimulating job							
5. I have access to structure and support through the decision support system							
6. I experience support from colleagues							
7. I experience support from management							
8. I experience insufficient opportunities to discuss and reflect with colleagues							
9. I experience insufficient support and appreciation from management							
10. Fatigue during long work shifts							
11. Lack of opportunities to influence my work							
12. Sedentary work that leads to physical strain							
13. Monotonous work							
14. Working in a production-driven organization with performance demands							
